# Newspaper Literature

**Published:** 1860-04

**Authors:** 


*Newspaper Literature.*—We call the attention of our readers to the following timely comments upon a great and growing evil. No one who has observed the course of the public press, can fail to recognize the justice of the reproof. It may be that these miserable sheets are preparing the way for something better. If so, God grant they may soon finish their work, and stand aside forever.

We copy from the Boston Medical and Surgical Journal.

"For a long time we have noticed with regret a marked change in the character of the contents of our daily papers. Some years since, certain matters were considered unfit for publication, except in the lowest journals, the presence of which in any respectable family would have been regarded as a disgrace. Now, however, it is almost impossible to find a newspaper which does not contain, upon every page, something which should never meet the eye of any member of a household.

"It was, therefore, with no little pleasure that we read some remarks upon this subject by Dr. Ray, in his report on the Butler Hospital for the

Insane. After calling attention to 'the unquestionable fact that insanity is increasing in all civilized communities,' he speaks of the influence of the law of imitation upon the human mind, and very justly remarks that—

"The slightest examination of this our modern life will show us a host of agencies belonging to the ordinary routine, which, by means of this law of sympathy and propensity to imitation, produce an unhealthy tone of feeling, which not only deranges the proper order and balance of the moral sentiments, but often terminates, at last, either in unequivocal disease, or in conduct where the element of moral depravity is mingled in some uncertain proportion with that of cerebral disorder and disease. In no age of the world, have these agencies been so effective or so numerous, as at the present. Far above and beyond all others is that of the press, whose power, during the last sixty years, has been extending at an unexampled rate. There is not a single phasis of human passion, not a single combination of its various elements, not a single development of its slumbering activities, not a single abnormal deviation from its ordinary channels, not a single manifestation of its effects on actual life, which is not displayed by the public press in the strongest colors which an ambitious rhetoric can give it. And thus, too, those sad and fearful chapters in human experience which, though filled with woe to the parties immediately concerned, once were scarcely known beyond the limits of a little community, are now presented to every reader in the land, with every circumstance that can add force or piquancy to the narrative. The columns of a single newspaper, without exaggeration, it may be said, contain more materials for stirring the sympathies of men, for good or for evil, than the unwritten lives of countless multitudes. They occupy the leisure moments of thousands, which would otherwise be given to listless rest, and furnish inexhaustible materials for thought or emotion—the only kind, perhaps, which they ever obtain. The ephemeral sheet which to-day is, and to-morrow is cast into the oven, goes forth on the wings of the wind, scattering its hetrogeneous influences upon every description of person. A murder or a suicide, a breach of trust or an audacious robbery, committed in the obscurest corner of the land, is proclaimed to all the world. The details of a disgusting criminal trial, exposing the darkest aspects of our nature, find an audience that no court-room less than a hemisphere could hold. * * * * *

"It is a common impression that the newspaper merely ministers to the natural curiosity of men to know what is passing around them; but it has another and a far more important effect. It is not every occurrence, whose communication to the world can be productive of unmingled good. For reasons just given, no small proportion of those which are thrust upon the reader's attention, leave a positively unhealthy impression; and when we consider that, besides the multitudes who, in addition to other reading, never pass a day without looking over a newspaper, there is a scarcely smaller number who read nothing else, we may get some faint idea of the magnitude of this result. The details of vice and crime which occupy so large a space in the daily sheet, repeated day after day, familiarize the mind with their hideous features, and thus blunt the edge of its finer sensibilities. The effect of it all is, that the mind not only becomes careless of moral distinctions, but incapable, in some degree, of perceiving them; its relish for the simply good and beautiful and true, is lost, and in its place we find an insatiable craving for what will create a strong sensation, and a positive sympathy, perhaps with wrong, and wrong-doers. By a well-known law of the animal economy, excessive activity of a function, leads at last, to a morbid condition of the organ; and thus it is that this kind of mental activity becomes a prolific source of cerebral dssorder—not of the more palpable forms, such as inflammation or softening, but of a degree of irritability or abnormal erythism which often terminates in overt disease."

"With the best of intentions, he partially exonerates the press in the following words:

"Far be it from me to lay the blame of all this mischief at the door of those who manage the newspaper-press. In responding to the call which is made for the kind of news in question, they believe, honestly, no doubt, that they are complying with a reasonable wish, and laboring worthily in their vocation. It is not for them, in exercising this function, to scrutinize too closely the remote and indirect effects of what they communicate, upon the minds of their readers. The fault really lies in the public taste which craves and demands such reading; and the true remedy consists, not in blaming the people connected with the press, or addressing to them philosophical reflections on the operations of the mind, but in refining and elevating the public taste, by improving our methods of education, and multiplying the means and appliances of a higher and sounder cultivation. This, certainly, is not very specific, and promises no immediate relief; but so it ever must be with all reforms that effect the exercise of the passions and propensities."

"We sincerely hope that such words, however kindly in their tone, may be regarded as a decided reflection upon the editorial corps. It is no compliment to tell them that they are always to *follow* and never *form* public taste; that they may legitimately vary their standard of morality in accordance with changes in public opinion; that, instead of using their almost unlimited power for the benefit of mankind, they may exert it in promoting the worst of causes, and be the most active agents in the demoralization of society.

"There are but three sources to which this great evil can be traced:—to a belief on the part of editors, that what they furnish is to be a real benefit to those who read; to negligence; or to the trading spirit, which is always ready to sell anything which the most morbid appetite may crave.

"That the first is true, we have not the credulity to believe; the second may happen occasionally, but will not explain the vitality and persistence so strongly manifested; the third is, probably, in most instances, the only correct solution.

"If the true remedy consists, as we are told above, 'in refining and elevating the public taste,' what more potent element can we call to our aid than this same public press, and, if this fails us, where shall we look for help? Those who thus have the power of purifying or corrupting the minds of the people should, we think, receive the first attention, and be taught, if they do not understand it now, the responsibility which rests upon them."

